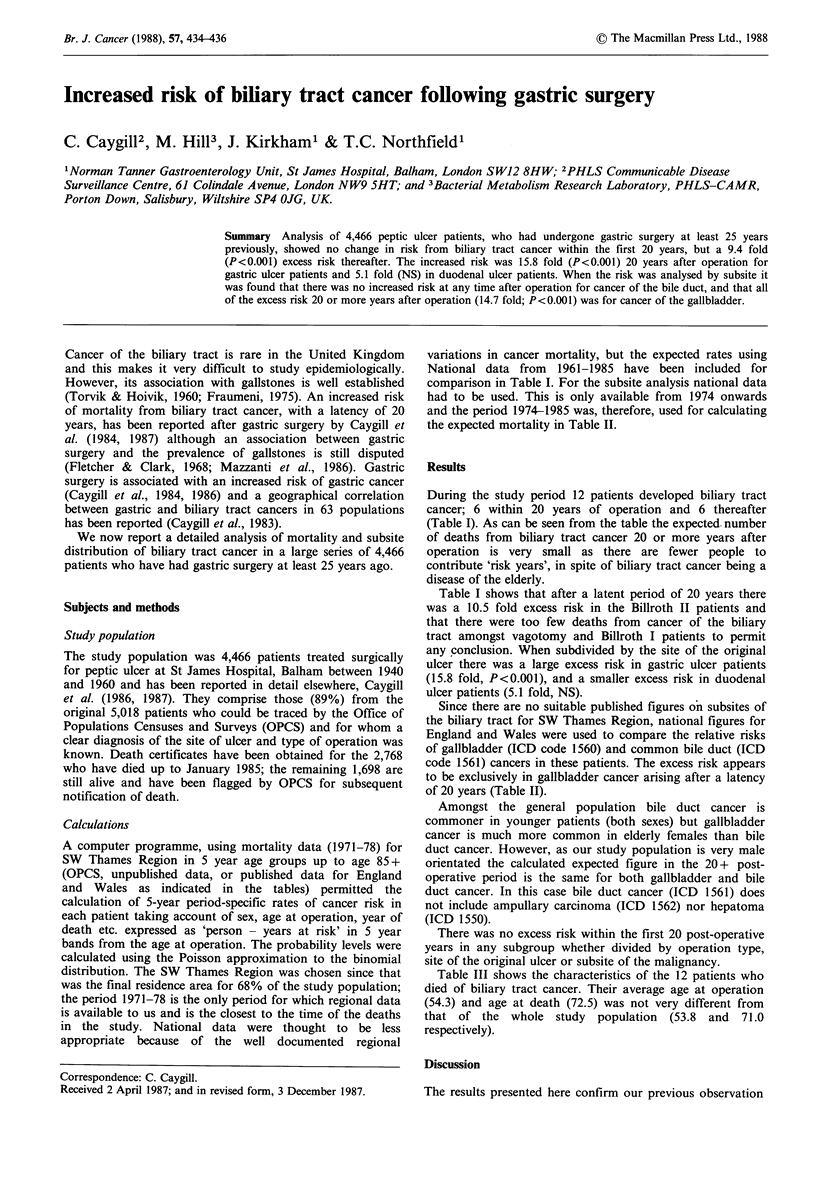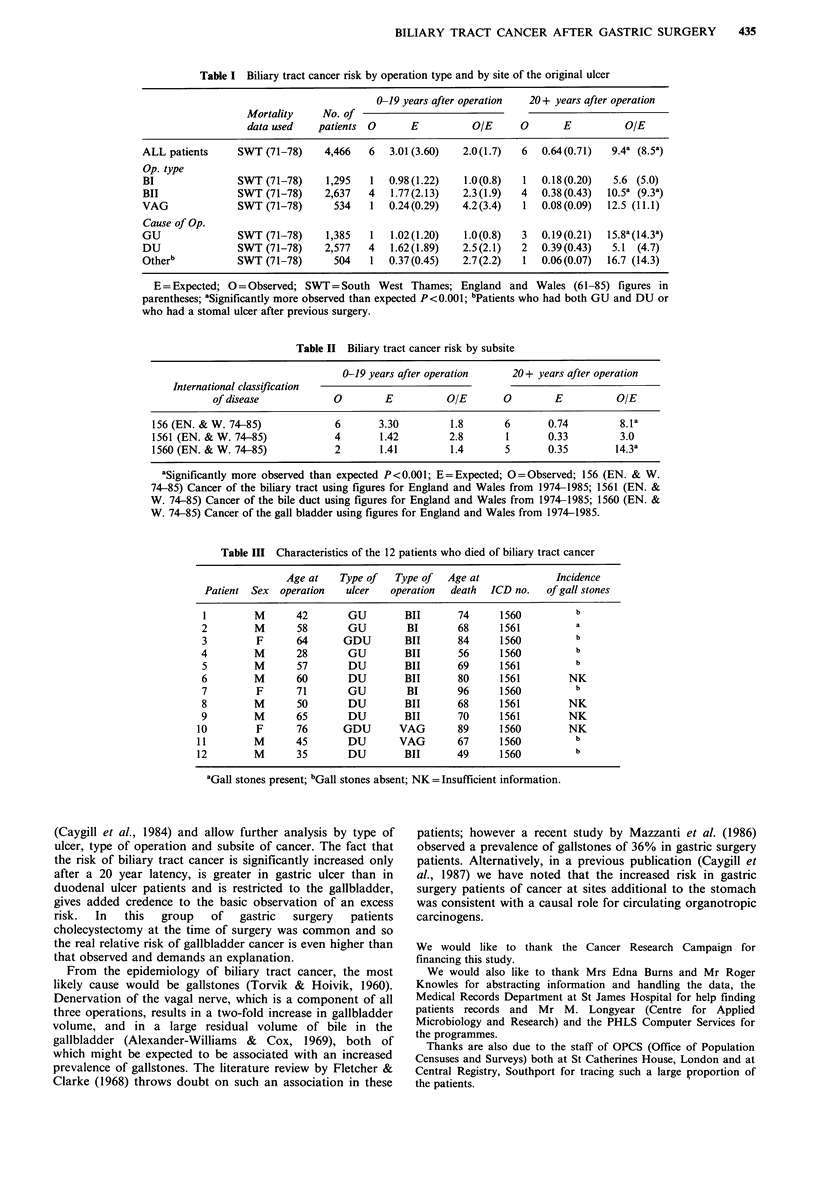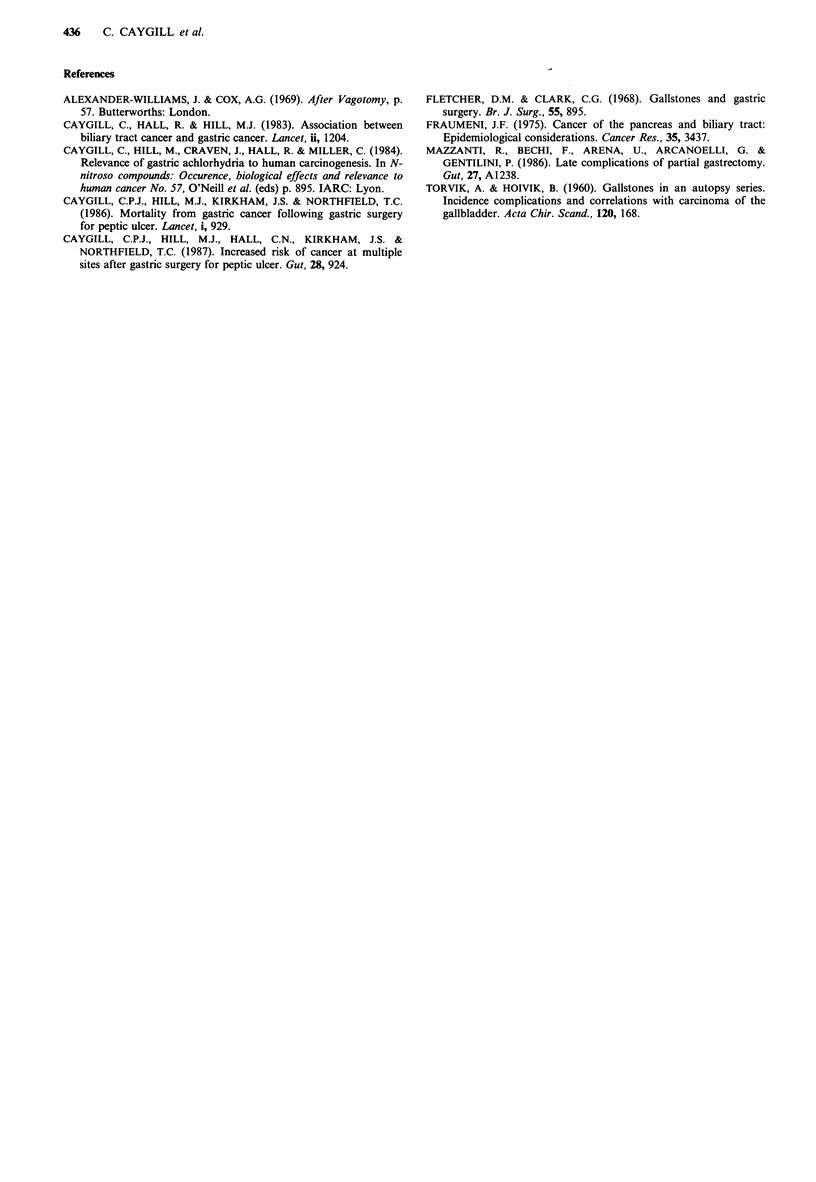# Increased risk of biliary tract cancer following gastric surgery.

**DOI:** 10.1038/bjc.1988.98

**Published:** 1988-04

**Authors:** C. Caygill, M. Hill, J. Kirkham, T. C. Northfield

**Affiliations:** Norman Tanner Gastroenterology Unit, St James Hospital, Balham, London, UK.

## Abstract

Analysis of 4,466 peptic ulcer patients, who had undergone gastric surgery at least 25 years previously, showed no change in risk from biliary tract cancer within the first 20 years, but a 9.4 fold (P less than 0.001) excess risk thereafter. The increased risk was 15.8 fold (P less than 0.001) 20 years after operation for gastric ulcer patients and 5.1 fold (NS) in duodenal ulcer patients. When the risk was analysed by subsite it was found that there was no increased risk at any time after operation for cancer of the bile duct, and that all of the excess risk 20 or more years after operation (14.7 fold; P less than 0.001) was for cancer of the gallbladder.


*Br. J. Cancer* (1988), **57**, 434–436 © The Macmillan Press Ltd., 1988

### Increased risk of biliary tract cancer following gastric surgery

C. Caygill[2], M. Hill[3], J. Kirkham[1] & T.C. Northfield[1]

[1]*Norman Tanner Gastroenterology Unit, St James Hospital, Balham, London SW12 8HW;* [2]*PHLS Communicable Disease Surveillance Centre, 61 Colindale Avenue, London NW9 5HT; and* [3]*Bacterial Metabolism Research Laboratory, PHLS–CAMR, Porton Down, Salisbury, Wiltshire SP4 0JG, UK.*

**Summary** Analysis of 4,466 peptic ulcer patients, who had undergone gastric surgery at least 25 years previously, showed no change in risk from biliary tract cancer within the first 20 years, but a 9.4 fold ($P<0.001$) excess risk thereafter. The increased risk was 15.8 fold ($P<0.001$) 20 years after operation for gastric ulcer patients and 5.1 fold (NS) in duodenal ulcer patients. When the risk was analysed by subsite it was found that there was no increased risk at any time after operation for cancer of the bile duct, and that all of the excess risk 20 or more years after operation (14.7 fold; $P<0.001$) was for cancer of the gallbladder.

Cancer of the biliary tract is rare in the United Kingdom and this makes it very difficult to study epidemiologically. However, its association with gallstones is well established (Torvik & Hoivik, 1960; Fraumeni, 1975). An increased risk of mortality from biliary tract cancer, with a latency of 20 years, has been reported after gastric surgery by Caygill *et al.* (1984, 1987) although an association between gastric surgery and the prevalence of gallstones is still disputed (Fletcher & Clark, 1968; Mazzanti *et al.*, 1986). Gastric surgery is associated with an increased risk of gastric cancer (Caygill *et al.*, 1984, 1986) and a geographical correlation between gastric and biliary tract cancers in 63 populations has been reported (Caygill *et al.*, 1983).

We now report a detailed analysis of mortality and subsite distribution of biliary tract cancer in a large series of 4,466 patients who have had gastric surgery at least 25 years ago.

### Subjects and methods

### *Study population*

The study population was 4,466 patients treated surgically for peptic ulcer at St James Hospital, Balham between 1940 and 1960 and has been reported in detail elsewhere, Caygill *et al.* (1986, 1987). They comprise those (89%) from the original 5,018 patients who could be traced by the Office of Populations Censuses and Surveys (OPCS) and for whom a clear diagnosis of the site of ulcer and type of operation was known. Death certificates have been obtained for the 2,768 who have died up to January 1985; the remaining 1,698 are still alive and have been flagged by OPCS for subsequent notification of death.

### *Calculations*

A computer programme, using mortality data (1971–78) for SW Thames Region in 5 year age groups up to age 85+ (OPCS, unpublished data, or published data for England and Wales as indicated in the tables) permitted the calculation of 5-year period-specific rates of cancer risk in each patient taking account of sex, age at operation, year of death etc. expressed as 'person – years at risk' in 5 year bands from the age at operation. The probability levels were calculated using the Poisson approximation to the binomial distribution. The SW Thames Region was chosen since that was the final residence area for 68% of the study population; the period 1971–78 is the only period for which regional data is available to us and is the closest to the time of the deaths in the study. National data were thought to be less appropriate because of the well documented regional variations in cancer mortality, but the expected rates using National data from 1961–1985 have been included for comparison in Table I. For the subsite analysis national data had to be used. This is only available from 1974 onwards and the period 1974–1985 was, therefore, used for calculating the expected mortality in Table II.

Correspondence: C. Caygill.
Received 2 April 1987; and in revised form, 3 December 1987.

### Results

During the study period 12 patients developed biliary tract cancer; 6 within 20 years of operation and 6 thereafter (Table I). As can be seen from the table the expected number of deaths from biliary tract cancer 20 or more years after operation is very small as there are fewer people to contribute 'risk years', in spite of biliary tract cancer being a disease of the elderly.

Table I shows that after a latent period of 20 years there was a 10.5 fold excess risk in the Billroth II patients and that there were too few deaths from cancer of the biliary tract amongst vagotomy and Billroth I patients to permit any conclusion. When subdivided by the site of the original ulcer there was a large excess risk in gastric ulcer patients (15.8 fold, $P<0.001$), and a smaller excess risk in duodenal ulcer patients (5.1 fold, NS).

Since there are no suitable published figures on subsites of the biliary tract for SW Thames Region, national figures for England and Wales were used to compare the relative risks of gallbladder (ICD code 1560) and common bile duct (ICD code 1561) cancers in these patients. The excess risk appears to be exclusively in gallbladder cancer arising after a latency of 20 years (Table II).

Amongst the general population bile duct cancer is commoner in younger patients (both sexes) but gallbladder cancer is much more common in elderly females than bile duct cancer. However, as our study population is very male orientated the calculated expected figure in the 20+ post-operative period is the same for both gallbladder and bile duct cancer. In this case bile duct cancer (ICD 1561) does not include ampullary carcinoma (ICD 1562) nor hepatoma (ICD 1550).

There was no excess risk within the first 20 post-operative years in any subgroup whether divided by operation type, site of the original ulcer or subsite of the malignancy.

Table III shows the characteristics of the 12 patients who died of biliary tract cancer. Their average age at operation (54.3) and age at death (72.5) was not very different from that of the whole study population (53.8 and 71.0 respectively).

### Discussion

The results presented here confirm our previous observation

**Table I** Biliary tract cancer risk by operation type and by site of the original ulcer

| | Mortality data used | No. of patients | 0–19 years after operation | | | 20+ years after operation | | |
|---|---|---|---|---|---|---|---|---|
| | | | O | E | O/E | O | E | O/E |
| ALL patients | SWT (71–78) | 4,466 | 6 | 3.01 (3.60) | 2.0 (1.7) | 6 | 0.64 (0.71) | 9.4[a] (8.5[a]) |
| *Op. type* | | | | | | | | |
| BI | SWT (71–78) | 1,295 | 1 | 0.98 (1.22) | 1.0 (0.8) | 1 | 0.18 (0.20) | 5.6 (5.0) |
| BII | SWT (71–78) | 2,637 | 4 | 1.77 (2.13) | 2.3 (1.9) | 4 | 0.38 (0.43) | 10.5[a] (9.3[a]) |
| VAG | SWT (71–78) | 534 | 1 | 0.24 (0.29) | 4.2 (3.4) | 1 | 0.08 (0.09) | 12.5 (11.1) |
| *Cause of Op.* | | | | | | | | |
| GU | SWT (71–78) | 1,385 | 1 | 1.02 (1.20) | 1.0 (0.8) | 3 | 0.19 (0.21) | 15.8[a] (14.3[a]) |
| DU | SWT (71–78) | 2,577 | 4 | 1.62 (1.89) | 2.5 (2.1) | 2 | 0.39 (0.43) | 5.1 (4.7) |
| Other[b] | SWT (71–78) | 504 | 1 | 0.37 (0.45) | 2.7 (2.2) | 1 | 0.06 (0.07) | 16.7 (14.3) |

E = Expected; O = Observed; SWT = South West Thames; England and Wales (61–85) figures in parentheses; [a]Significantly more observed than expected $P < 0.001$; [b]Patients who had both GU and DU or who had a stomal ulcer after previous surgery.

**Table II** Biliary tract cancer risk by subsite

| International classification of disease | 0–19 years after operation | | | 20+ years after operation | | |
|---|---|---|---|---|---|---|
| | O | E | O/E | O | E | O/E |
| 156 (EN. & W. 74–85) | 6 | 3.30 | 1.8 | 6 | 0.74 | 8.1[a] |
| 1561 (EN. & W. 74–85) | 4 | 1.42 | 2.8 | 1 | 0.33 | 3.0 |
| 1560 (EN. & W. 74–85) | 2 | 1.41 | 1.4 | 5 | 0.35 | 14.3[a] |

[a]Significantly more observed than expected $P < 0.001$; E = Expected; O = Observed; 156 (EN. & W. 74–85) Cancer of the biliary tract using figures for England and Wales from 1974–1985; 1561 (EN. & W. 74–85) Cancer of the bile duct using figures for England and Wales from 1974–1985; 1560 (EN. & W. 74–85) Cancer of the gall bladder using figures for England and Wales from 1974–1985.

**Table III** Characteristics of the 12 patients who died of biliary tract cancer

| Patient | Sex | Age at operation | Type of ulcer | Type of operation | Age at death | ICD no. | Incidence of gall stones |
|---|---|---|---|---|---|---|---|
| 1 | M | 42 | GU | BII | 74 | 1560 | [b] |
| 2 | M | 58 | GU | BI | 68 | 1561 | [a] |
| 3 | F | 64 | GDU | BII | 84 | 1560 | [b] |
| 4 | M | 28 | GU | BII | 56 | 1560 | [b] |
| 5 | M | 57 | DU | BII | 69 | 1561 | [b] |
| 6 | M | 60 | DU | BII | 80 | 1561 | NK |
| 7 | F | 71 | GU | BI | 96 | 1560 | [b] |
| 8 | M | 50 | DU | BII | 68 | 1561 | NK |
| 9 | M | 65 | DU | BII | 70 | 1561 | NK |
| 10 | F | 76 | GDU | VAG | 89 | 1560 | NK |
| 11 | M | 45 | DU | VAG | 67 | 1560 | [b] |
| 12 | M | 35 | DU | BII | 49 | 1560 | [b] |

[a]Gall stones present; [b]Gall stones absent; NK = Insufficient information.

(Caygill *et al.*, 1984) and allow further analysis by type of ulcer, type of operation and subsite of cancer. The fact that the risk of biliary tract cancer is significantly increased only after a 20 year latency, is greater in gastric ulcer than in duodenal ulcer patients and is restricted to the gallbladder, gives added credence to the basic observation of an excess risk. In this group of gastric surgery patients cholecystectomy at the time of surgery was common and so the real relative risk of gallbladder cancer is even higher than that observed and demands an explanation.

From the epidemiology of biliary tract cancer, the most likely cause would be gallstones (Torvik & Hoivik, 1960). Denervation of the vagal nerve, which is a component of all three operations, results in a two-fold increase in gallbladder volume, and in a large residual volume of bile in the gallbladder (Alexander-Williams & Cox, 1969), both of which might be expected to be associated with an increased prevalence of gallstones. The literature review by Fletcher & Clarke (1968) throws doubt on such an association in these patients; however a recent study by Mazzanti *et al.* (1986) observed a prevalence of gallstones of 36% in gastric surgery patients. Alternatively, in a previous publication (Caygill *et al.*, 1987) we have noted that the increased risk in gastric surgery patients of cancer at sites additional to the stomach was consistent with a causal role for circulating organotropic carcinogens.

We would like to thank the Cancer Research Campaign for financing this study.

We would also like to thank Mrs Edna Burns and Mr Roger Knowles for abstracting information and handling the data, the Medical Records Department at St James Hospital for help finding patients records and Mr M. Longyear (Centre for Applied Microbiology and Research) and the PHLS Computer Services for the programmes.

Thanks are also due to the staff of OPCS (Office of Population Censuses and Surveys) both at St Catherines House, London and at Central Registry, Southport for tracing such a large proportion of the patients.

### References

ALEXANDER-WILLIAMS, J. & COX, A.G. (1969). *After Vagotomy*, p. 57. Butterworths: London.

CAYGILL, C., HALL, R. & HILL, M.J. (1983). Association between biliary tract cancer and gastric cancer. *Lancet*, **ii**, 1204.

CAYGILL, C., HILL, M., CRAVEN, J., HALL, R. & MILLER, C. (1984). Relevance of gastric achlorhydria to human carcinogenesis. In *N-nitroso compounds: Occurence, biological effects and relevance to human cancer No. 57*, O'Neill *et al.* (eds) p. 895. IARC: Lyon.

CAYGILL, C.P.J., HILL, M.J., KIRKHAM, J.S. & NORTHFIELD, T.C. (1986). Mortality from gastric cancer following gastric surgery for peptic ulcer. *Lancet*, **i**, 929.

CAYGILL, C.P.J., HILL, M.J., HALL, C.N., KIRKHAM, J.S. & NORTHFIELD, T.C. (1987). Increased risk of cancer at multiple sites after gastric surgery for peptic ulcer. *Gut*, **28**, 924.

FLETCHER, D.M. & CLARK, C.G. (1968). Gallstones and gastric surgery. *Br. J. Surg.*, **55**, 895.

FRAUMENI, J.F. (1975). Cancer of the pancreas and biliary tract: Epidemiological considerations. *Cancer Res.*, **35**, 3437.

MAZZANTI, R., BECHI, F., ARENA, U., ARCANOELLI, G. & GENTILINI, P. (1986). Late complications of partial gastrectomy. *Gut*, **27**, A1238.

TORVIK, A. & HOIVIK, B. (1960). Gallstones in an autopsy series. Incidence complications and correlations with carcinoma of the gallbladder. *Acta Chir. Scand.*, **120**, 168.